# Chondroitin polymerizing factor promotes breast carcinoma cell proliferation, invasion and migration and affects expression of epithelial‐mesenchymal transition‐related markers

**DOI:** 10.1002/2211-5463.13062

**Published:** 2021-01-07

**Authors:** Yang Li, Hui Gong, Lei Feng, Dan Mao, Yujie Xiao, Yunqi Wang, Lizhong Huang

**Affiliations:** ^1^ Department of Traditional Chinese Medicine /Integrative Oncology Hunan Cancer Hospital/The Affiliated Cancer Hospital of Xiangya School of Medicine Central South University Changsha China; ^2^ Department of Medical Oncology Hunan Academy of Traditional Chinese Medicine Affiliated Hospital Changsha China; ^3^ Department of Integrated Traditional Chinese and Western Medicine The Second Xiangya Hospital Central South University Changsha China; ^4^ College of Integrated Chinese and Western Medicine Hunan University of Chinese Medicine Changsha China

**Keywords:** breast carcinoma, CHPF, EMT, invasion, migration, viability

## Abstract

Chondroitin polymerizing factor (CHPF) plays an important role in the development of certain malignant tumors. However, the role of CHPF in breast carcinoma (BRCA) and its underlying mechanism are still not fully elucidated. Expression profiles for CHPF in BRCA tissues were retrieved from The Cancer Genome Atlas database and used for prognostic analysis. Cell viability, invasion and migration were measured *in vitro* using MCF7 and MDA‐MB‐231 cell lines upon knockdown or over‐expression of CHPF. Bioinformatic analysis showed that CHPF was substantially upregulated in BRCA tissues, and a quantitative reverse transcriptase‐PCR was performed to confirm its upregulation in BRCA cells. High expression of CHPF was observed to be significantly associated with pathologic stage, metastasis and worse prognosis. We also observed that depletion of CHPF significantly inhibited cell proliferative, invasive and migratory abilities, whereas overexpression of CHPF exerted the opposite effects. Furthermore, analysis of the GEPIA database revealed that CHPF expression is positively correlated with the epithelial–mesenchymal transition‐related markers vimentin, Snail, Slug and motion‐related protein matrix metallopeptidase 2; these findings were confirmed via western blotting. Our data suggest that CHPF may contribute to BRCA progression by modulating epithelial–mesenchymal transition‐related markers and matrix metallopeptidase 2 expression.

## Graphical abstract

Here, we found that chondroitin polymerizing factor is upregulated in breast carcinoma and associated with worse prognosis according to bioinformatic analysis. Functionally, chondroitin polymerizing factor acts as an oncogene that promotes breast carcinoma cell progression by facilitating proliferation, invasion and migration, as well as by elevating the expression of matrix metallopeptidase 2 and epithelial–mesenchymal transition‐related markers.

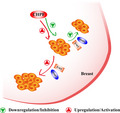

**Abbreviations**
ANOVA: one‐way analysis of variance
BRCA: breast carcinoma
CCK‐8: cell counting kit‐8
CHPF: chondroitin polymerizing factor
CS: chondroitin sulfate
DFS: disease‐free survival
DSS: disease‐specific survival
ECM: extracellular matrix
EMT: epithelial‐mesenchymal transition
GAPDH: glyceraldehyde‐3‐phosphate dehydrogenase
GEPIA: gene expression profiling interactive analysis
GSEA: gene set enrichment analysis
MMP2: matrix metallopeptidase 2
PFS: progress‐free survival
qRT‐PCR: quantitative reverse transcriptase‐PCR
siRNA: small interfering RNA
TCGA: The Cancer Genome Atlas

Breast carcinoma (BRCA) is the most commonly diagnosed cancer in women and ranks the second among causes of cancer relative death in women [1]. Globally, the incidence rate of BRCA has been rising rapidly over recent decades [2]. It is reported to account for 30% of all new cancer diagnoses in women [3]. New therapies have improved the survival of patients. However, 25–50% of patients with early‐stage BRCA eventually develop metastatic BRCA [4]. Tumor metastasis is a major obstacle to the treatment of BRCA [5] and so the search for key molecules that regulate tumor cell metastasis is very important with respect to improving the prognosis of BRCA and developing new therapeutic strategies.

Chondroitin sulfate (CS) is the main component of extracellular matrix (ECM) and serves as a scaffold for the structural integrity of tissues [6]. It is a linear polysaccharide composed of repeating disaccharide units of *N*‐acetyl‐d‐galactosamine and d‐glucuronic acid residues, which covalently bind proteins, making them proteoglycans [7, 8]. The biosynthesis of intact CS is a stepwise process involving multiple enzymes [7]. Six members of CS family have been identified, including human chondroitin polymerizing factor (CHPF). CHPF is a type of glycosyltransferase used for chondroitin synthesis [9] and can interact with other members to regulate the extension of CS chain [10]. Furthermore, knockdown of CHPF leads to specific elimination of CS and dermatan sulfate [11]. Thus, regulation of CHPF expression is essential for CS biosynthesis and proteoglycan production.

Increasing evidence indicates that CS participates in tumor progression and metastasis [12]. Specifically, CS is highly expressed in head and neck squamous cell carcinoma and hepatocellular carcinoma [13, 14]. Therefore, it is reasonable to assume that CHPF may play an important role in the occurrence and development of tumors. Currently, CHPF has been reported to be upregulated in lung adenocarcinoma, non‐small‐cell lung cancer, gliomas and malignant melanoma, etc. [15, 16, 17, 18], suggesting that it plays an important role in tumor progression. However, as far as we know, the expression of CHPF in BRCA and its role in tumor progression are not clear.

In the present study, we aimed to explore the abnormal expression of CHPF in BRCA based on The Cancer Genome Atlas (TCGA) database and assess its prognostic value. Furthermore, we evaluated its effects on the phenotype of the BRCA cell. In addition, correlation between CHPF and motion‐related molecules was also explored. The results obtained demonstrate that CHPF is highly expressed in BRCA, and may act as a cancer promoter to increase cell viability and invasiveness by regulating matrix metallopeptidase 2 (MMP2) expression and epithelial‐mesenchymal transition (EMT)‐related proteins.

## Materials and methods

### Public data collection and analysis

From TCGA database (https://cancergenome.nih.gov), we downloaded the mRNA‐sequencing dataset of BRCA, including 1109 BRCA cases and 113 normal adjacent breast cases. The ‘edgeR’ package was used for the normalization of mRNA‐sequencing data [19]. *P* < 0.05 was considered statistically significant. Differentially expressed genes were accepted with a fold change > 1.5.

The Oncomine database (https://www.oncomine.org) is an online cancer microarray database and web‐based data‐mining platform. Differences in CHPF with respect to invasive BRCA and normal adjacent tissues were retrieved from TCGA Breast Statistics in the Oncomine platform, including 532 invasive BRCA and 61 paired normal breast tissues. The gene expression profile was measured experimentally using the Agilent mRNA expression microarrays platform (A_23_P131288, https://www.agilent.com).

For survival analysis, mRNA expression of CHPF in patients with BRCA was assessed based on the Kaplan–Meier plotter database (http://kmplot.com/analysis). Patients with complete clinical data obtained from TCGA database were divided into high and low expression of CHPF groups using the median CHPF expression value as criteria. Moreover, we adopted chi‐squared test to assess the correlation between CHPF and clinical symptoms of BRCA. Cox regression analysis was carried out to investigate the relationship between overall survival and clinicopathologic characteristics of patients using spss, version 22.0 (IBM Corp., Armonk, NY, USA).

The Gene Expression Profiling Interactive Analysis (GEPIA) database (http://gepia.cancer‐pku.cn) is a newly developed web server for interaction analysis, and provides correlation analysis [20]. We adopted this database to determine the significant correlation between CHPF and motion‐related genes in BRCA. The Pearson score was used to determine the correlation coefficient.

Gene set enrichment analysis (GSEA) was performed to identify the possible pathways related to CHPF using GSEA software (http://software.broadinstitute.org/gsea/index.jsp) [21]. *P* < 0.05 with a false discovery rat (FDR *q*‐val) < 0.25 was considered statistically significant.

### Cell culture and transfection

The BRCA cell lines MDA‐MB‐231, SK‐BR‐3 and MCF7, as well as normal epithelial cell line MCF‐10A, were obtained from the Cell Resource Center, Shanghai Institute of Life Sciences, Chinese Academy of Sciences (Shanghai, China) and incubated in Roswell Park Memorial Institute‐1640 (RPMI‐1640) (Procell, Wuhan, China) medium containing 10% fetal bovine serum at 37 °C with 5% CO_2._ Additionally, cells were supplemented with 100 U·mL^−1^ penicillin and 0.1 mg·mL^−1^ streptomycin.

Cells were transfected with small interfering RNAs (siRNAs; CHPF depletion, CHPF‐siRNA1: 5'‐AGCTGGCCATGCTACTCTTTG‐3', CHPF‐siRNA2: 5'‐TGAATGGCTACCGACGCTTTG ‐3') or pcDNA3.1‐CHPF (CHPF overexpression) by using Lipofectamine 2000 kit (Invitrogen, Carlsbad, CA, USA) in accordance with the the manufacturer's instructions. Scramble siRNA (si‐con: 5'‐CGAACTCACTGGTCTGACC‐3') or pcDNA3.1 empty vector served as control. The CHPF‐siRNAs and pcDNA3.1‐CHPF were synthesized by the GenePharma Co., Ltd (Shanghai, China).

### Quantitative reverse transcriptase‐PCR (qRT‐PCR)

Total RNAs were extracted from cells using TRIzol reagent (Invitrogen) and reverse transcribed to cDNA using the PrimeScript RT Reagent Kit (TaKaRa, Dalian, China). The mRNA level was detected by a SYBR Premix Ex Taq system (TaKaRa). The primers were:

CHPF forward, 5'‐AACGCACGTACCAGGAGATCCA‐3';

CHPF reverse, 5'‐GGATGGTGCTGGAATACCCACG‐3';

GAPDH forward, 5'‐TGTGTCCGTCGTGGATCTGA‐3';

GAPDH reverse, 5'‐CCTGCTTCACCACCTTCTTGA‐3'.

Glyceraldehyde‐3‐phosphate dehydrogenase (GAPDH) served as a reference. Relative levels of individual gene mRNA transcripts were reckoned using the comparative quantification cycle (Cq) method (2^−ΔΔCq^).

### Western blotting

After transfection for 24 h, cells were lysed in the RIPA lysis buffer (Beyotime, Nantong, China) on ice. Protein from each sample (20 μg protein each hole) was separated by using SDS/PAGE and transferred onto poly(vinylidene difluoride) membranes. Subsequently, these membranes were blocked for 1 h with 5% skimmed milk powder and incubated with specific primary and secondary antibodies. An enhanced electrochemiluminescence kit (Beyotime) was performed for signal development. The data were quantified using imagej (NIH, Bethesda, MD, USA). The primary antibodies used were: anti‐CHPF (PA5‐50009), anti‐vimentin (MA5‐16409), anti‐Snail (PA5‐23472), anti‐Slug (PA1‐86737) and anti‐MMP2 (PA5‐85197), and anti‐GAPDH (PA1‐988). All of the primary antibodies and secondary antibodies were acquired from Thermo Fisher Scientific (Waltham, MA, USA).

### Cell proliferation assay

After transfection for 24 h, cells were digested to prepare the cell suspension. A cell counting Kit‐8 (CCK‐8) kit was used to determine cell proliferation. Briefly, approximately 1000 cells per well were plated into 96‐well plates. CCK‐8 reagent (10 μL) was supplied to each well at the designated time point of 0, 24, 48 and 72 h, and the cells were maintained at 37 °C for another 1.5 h. The absorbance value was tested at 450 nm.

For the clone formation assay, approximately 400 cells were plated into a 60‐mm dish containing 5 mL of medium, and cultured at 37 °C in a cell incubator for 1–2 weeks. Following the formation of sufficiently large clones, cells were immobilized with 5 mL of 4% paraformaldehyde for 30 min and then stained with 0.1% crystal violet for 30 min. Finally, the colonies was photographed and counted.

### Cell invasion and migration assay

A transwell assay was adopted to assess cell invasive and migratory capacities. For the invasion assay, upper chambers were coated with Matrigel (Corning Inc., Corning, NY, USA) (1 : 6 dilution of serum‐free medium, 100 μL). After transfection for 24 h, cells were suspended in serum‐free medium and added into the coated upper chamber at a density of 1 × 10^4^ cells per well. The lower chambers were supplied with 500 μL of complete medium. After incubating for 24 h, the invasive cells were immobilized with 4% paraformaldehyde and stained with 0.1% crystal violet. Then, cells were photographed and counted under a microscope. The experimental procedure of the migration assay is similar to that of the invasion assaym except that Matrigel was absent and 5 × 10^3^ cells were added to the upper chamber.

### Statistical analysis

Data are presented as the mean ± SD and were analyzed using prism, version 5.0 (GraphPad Software Inc., San Diego, CA, USA). All of the experiments were repeated three times independently. *P* < 0.05 was considered statistically significant. Comparisons between two groups were analyzed using Student's *t*‐test. One‐way analysis of variance (ANOVA) was used to compare multiple groups followed by a Bonferroni post‐hoc test. Correlation analyses were conducted using the Pearson correlation coefficient.

## Results

### CHPF expression is elevated in BRCA

**Fig. 1:**
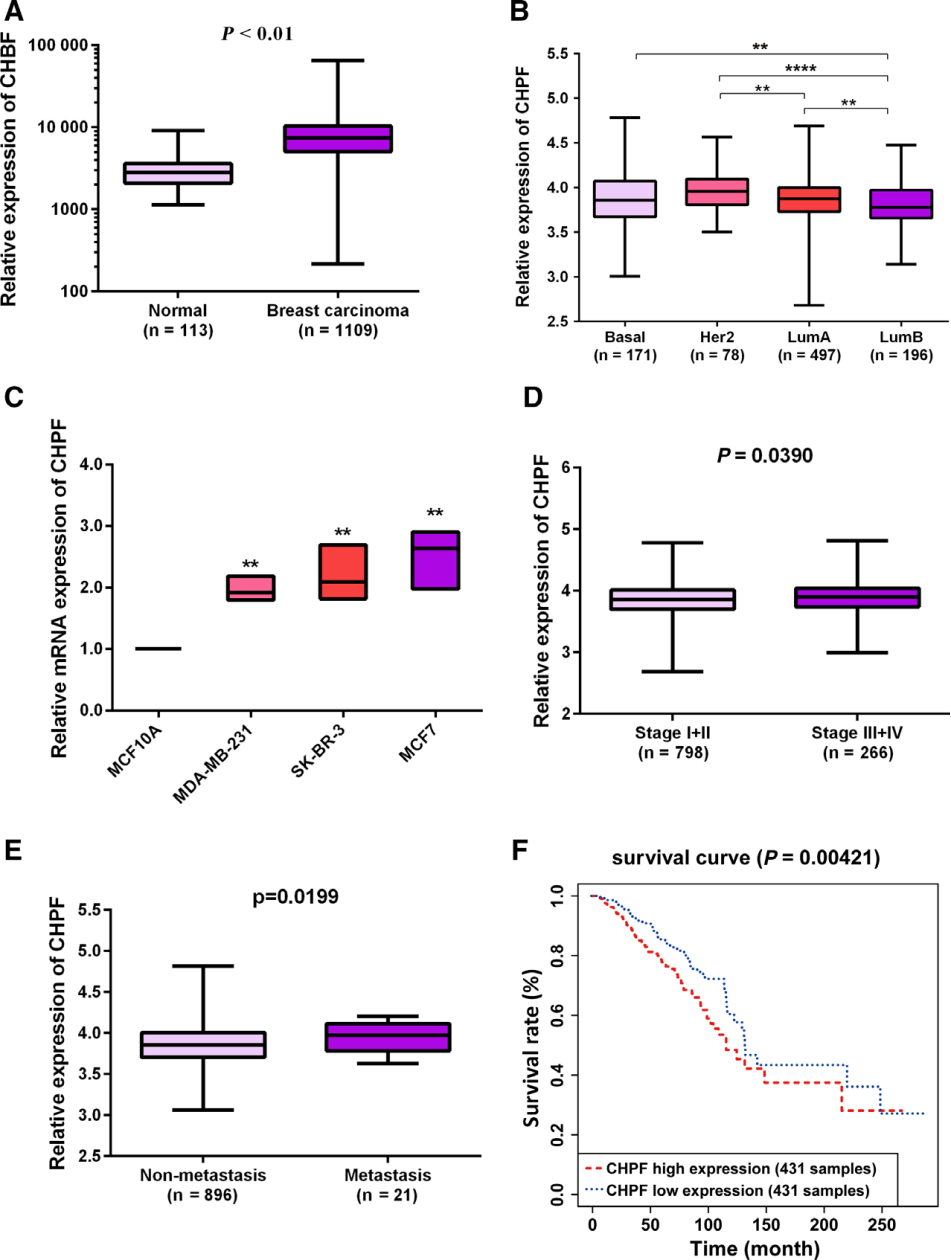
CHPF expression and survival curve. (A) High levels of CHPF were uncovered by bioinformatics analysis based on TCGA database. *P* < 0.01 vs. normal adjacent breast tissues. (B) CHPF expression in different subtypes. **P < 0.01, ****P < 0.0001. (C) mRNA levels of CHPF were measured by qRT‐PCR in three BRCA cell lines (MCF7, MDA‐MB‐231 and SK‐BR‐3) and human mammary epithelial cells (MCF‐10A). Data are presented as the mean ± SD for three independent experiments, **P < 0.01 vs. MCF‐10A. (D) CHPF expression in stage I + II vs. stage III + IV. (E) CHPF expression in non‐metastatic BRCA cases and metastatic BRCA cases. (F) The survival curve was plotted via the Kaplan–Meier method based on TCGA database using a log‐rank test for comparison. Statistical analyses in (A), (D) and (E) were analyzed using Student's *t*‐test. Statistical analyses in (B) and (C) were analyzed using ANOVA followed by a Bonferroni post‐hoc test.

We first analyzed the mRNA levels of CHPF in human BRCA based on TCGA database and observed a significant increase of CHPF expression in tumor tissues compared to normal adjacent breast tissues (*P* < 0.01) (Fig. 1A). We also found that the transcript expression of CHPF was notably elevated in invasive BRCA tissues compared to normal adjacent breast tissues (*P* < 0.01) (Fig. S1). Moreover, we retrieved four subtypes of BRCA according to TCGA clinical data and found that CHPF expression was significantly increased in the Her2 subtype compared to LumA and LumB subtypes, and highly expressed in Basal and LumA subtypes compared to the LumB subtype (*P* < 0.01) (Fig. 1B).

To confirm CHPF expression in BRCA, we selected three BRCA cell lines, MDA‐MB‐231, SK‐BR‐3 and MCF7, to detect mRNA levels of CHPF. Normal epithelial breast cell line MCF10A served as a control. Similar to previous findings, CHPF was upregulated in all BRCA cell lines compared to the control (*P* < 0.01) (Fig. 1C). All of these results suggest that CHPF is upregulated in human BRCA and may play a role in the progression of BRCA.

### CHPF is associated with poor prognosis in BRCA patients

To evaluate the prognostic values of CHPF in BRCA, we first adopted chi‐square tests to assess the correlation between CHPF and clinical factors. The data presented in Table 1 indicated that CHPF expression had significant correlations with pathologic stage (*P* = 0.016) and pathologic‐metastasis (M) (*P* = 0.002). Meanwhile, we also obtained the same results using TCGA data with standardized processing, indicating that CHPF expression was highly expressed in stage III + IV vs. stage I + II and also highly expressed in metastatic BRCA cases vs. non‐metastatic BRCA cases, with significant differences (Fig. 1D,E). Moreover, the survival curve obtained via the Kaplan–Meier method showed that patients with high levels of CHPF presented a worse survival compared to patients with low CHPF expression (*P* < 0.01) (Fig. 1F). In Furthermore, high expression of CHPF resulted in a poor disease‐free survival (DFS), disease‐specific survival (DSS) and progress‐free survival (PFS) compared to the low CHPF expression group (*P* < 0.05) (Fig. S2A–C). Next, we performed Cox regression analysis to assess the prognostic values of CHPF. Univariate analysis demonstrated that CHPF expression, pathologic stage, ‐tumor (T), ‐M and ‐node (N), and age were all related to the overall survival for patients with BRCA (*P* < 0.05) (Table 2). These candidate parameters were further applied in multivariate analysis to identify independent prognosticators for survival. The data indicated that pathologic stage, pathologic‐M and age could be regarded as independent prognosticators of BRCA patients (*P* < 0.05) (Table 2). Collectively, these findings suggest that CHPF may be related to a poor prognosis for patients with BRCA.

**Table 1:** Correlation between clinical characteristics and CHPF expression in BRCA patients based on the RNA‐sequencing dataset from TCGA.

Characteristics	Expression of CHPF		*P* value
	Low	High	
Age (years)			
< 60	236	241	0.732
≥ 60	195	190	
Gender			
Female	427	424	0.363
Male	4	7	
Pathologic stage			
I + II	344	314	0.016*
III + IV	87	117	
Pathologic T			
T1 + T2	366	371	0.629
T3 + T4	65	60	
Pathologic M			
M0	429	417	0.002*
M1	2	14	
Pathologic N			
N0	223	200	0.117
N1 + N2 + N3	208	231	

* *P* < 0.05.

**Table 2:** Cox regression analysis for prognosticators in BRCA patients based on TCGA database. CI, confidence interval; HR, hazard ratio.

Variables	Univariate analysis			Multivariate analysis		
	*P*‐value	HR	95% CI	*P*‐value	HR	95% CI
CHPF expression (high/low)	0.016*	1.541	1.083–2.194	0.084	1.375	0.958–1.972
Pathologic stage (I + II/III + IV)	0.000*	2.789	1.953–3.983	0.023*	1.913	1.092–3.353
Pathologic‐T (T1 + T2/T3 + T4)	0.001*	1.920	1.293–2.851	0.824	0.943	0.562–1.582
Pathologic M (M0/M1)	0.000*	6.362	3.569–11.339	0.010*	2.384	1.236–4.598
Pathologic N (N0/N1 + N2 + N3)	0.000*	2.133	1.471–3.093	0.055	1.563	0.990–2.466
Age (< 60/≥ 60)	0.000*	2.002	1.402–2.859	0.000*	2.032	1.407–2.935
Gender (female/male)	0.877	0.856	0.119–6.137			

* *P *< 0.05.

### CHPF affects BRCA cell proliferation, invasion and migration

**Fig. 2:**
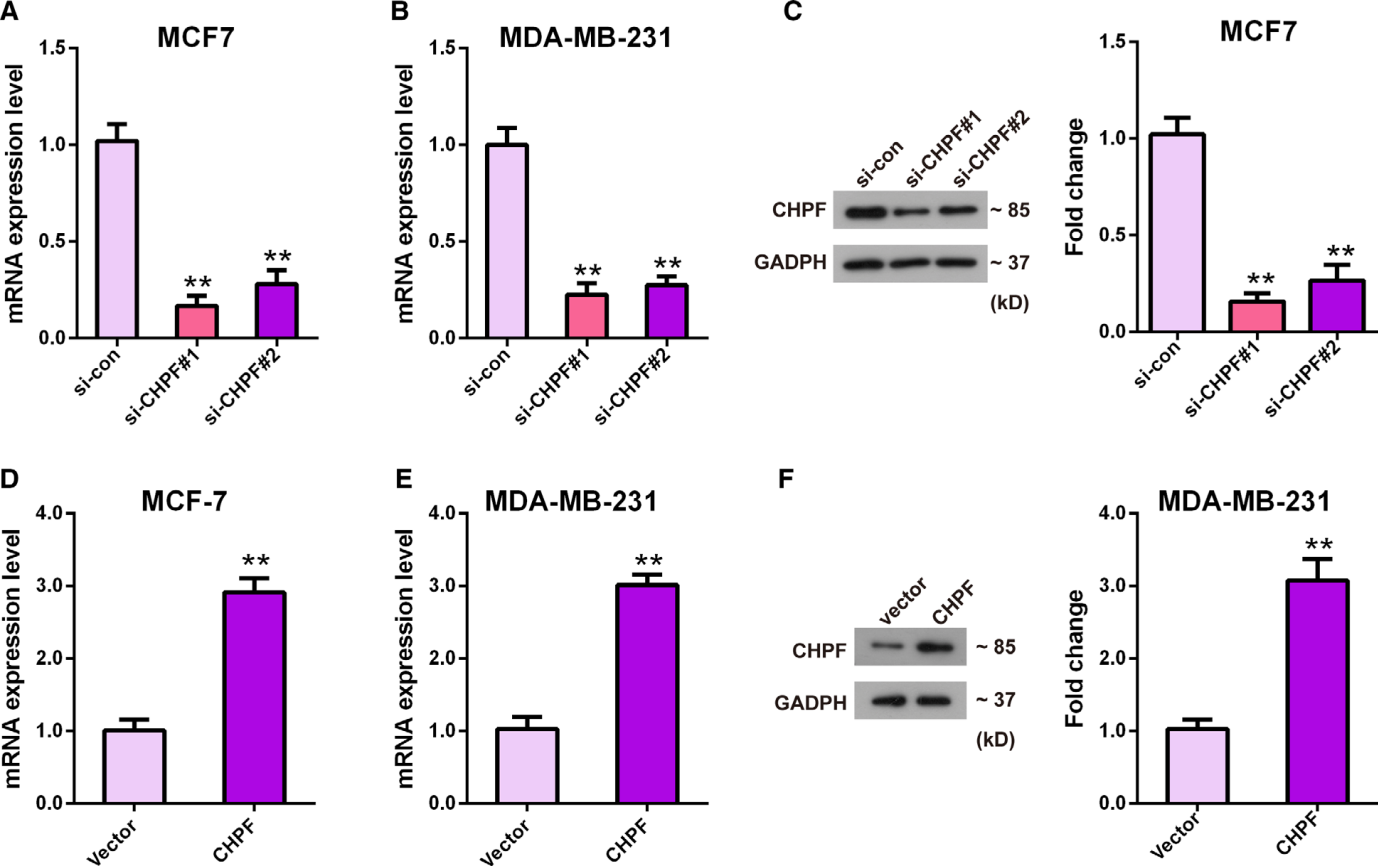
Detection of knockdown and overexpression of CHPF efficiency. qRT‐PCR (A, B) and western blotting (C) were used to test mRNA (A, B) and protein (C) levels of CHPF after transfection with si‐CHPF#1, si‐CHPF#2 and si‐con. qRT‐PCR (D, E) and western blotting (F) were used to test mRNA (D, E) and protein (F) levels of CHPF after transfection with pcDNA3.1‐CHPF and pcDNA3.1. **P < 0.01 vs. si‐con group or vector group. Data are presented as the mean ± SD for three independent experiments. Statistical analyses in (A), (B) and (C) were performed by ANOVA followed by a Bonferroni post‐hoc test. Statistical analyses in (D), (E) and (F) were analyzed using Student's *t*‐test.

To assess the effect of CHPF on cell biological behaviors of BRCA, we conducted CCK‐8, clone formation and transwell assays. qRT‐PCR analysis served to determine the mRNA levels of CHPF after altering its expression in MCF‐7 and MDA‐MB‐231 cells. The protein levels of CHPF were determined by western blotting. The results shown in Fig. 2 indicated that CHPF‐depleted MCF7 cells and CHPF‐enhanced MDA‐MB‐231 cells were all successfully conducted (*P* < 0.01). In view of the si‐CHPF#1 group presenting a better knockdown efficiency compared to the si‐CHPF#2 group, si‐CHPF#1 was selected for performing the following knockdown tests, and was represented as si‐CHPF.

**Fig. 3:**
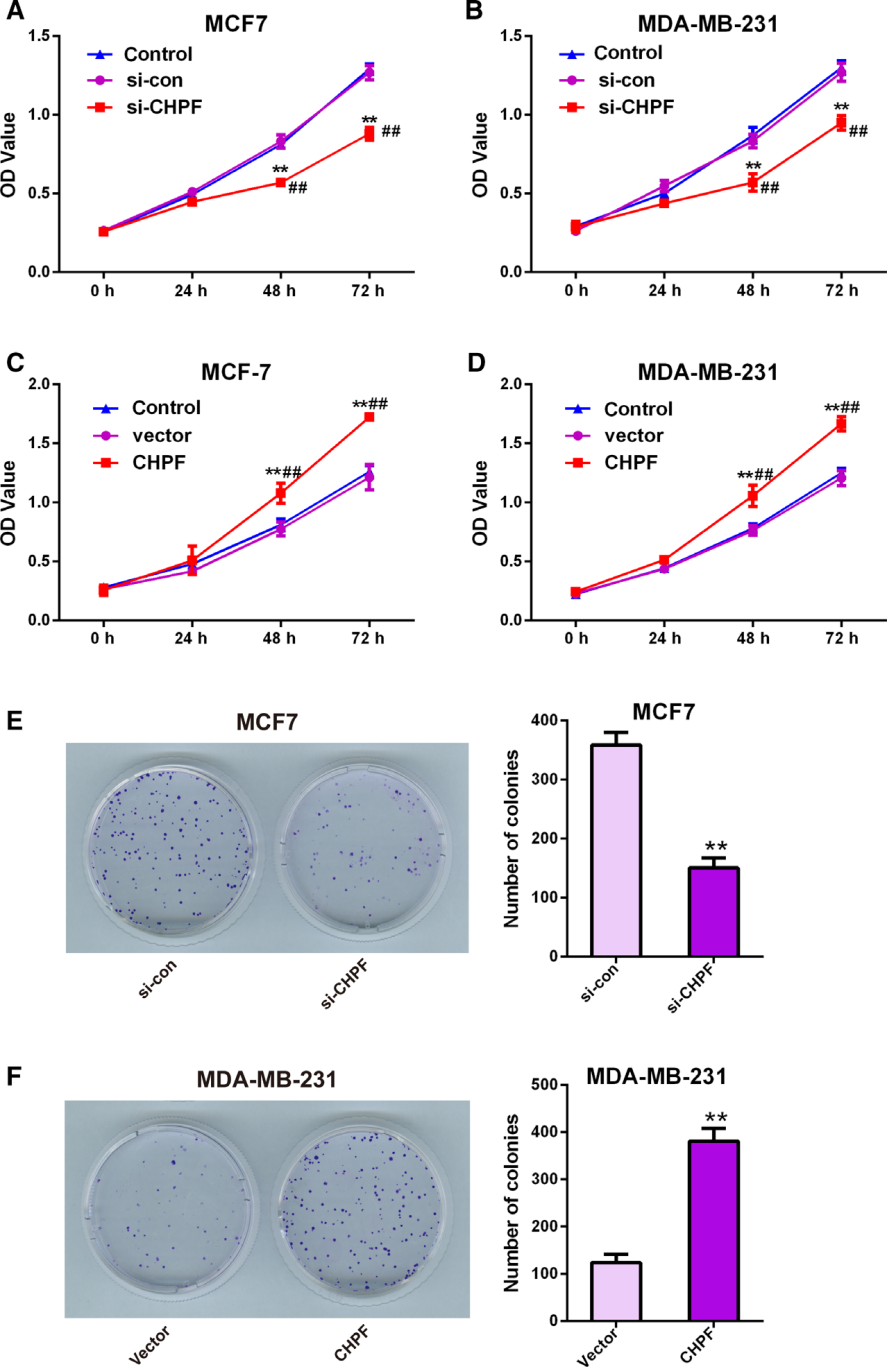
CHPF expression affected BRCA cell proliferation. A CCK‐8 assay was used to determine BRCA cell proliferation. MCF7 (A, C) and MDA‐MB‐231 (B,D) cells were transfected with si‐CHPF, si‐con and control, or pcDNA3.1‐CHPF and pcDNA3.1. A clone formation assay (E, F) was used to detect MCF7 (E) and MDA‐MB‐231 (F) Cell viability after transfection with si‐CHPF and si‐con (E) or pcDNA3.1‐CHPF and pcDNA3.1 (F). **P < 0.01 vs. si‐con group or vector group. ##*P* < 0.01 vs. control group. Data are presented as the mean ± SD for three independent experiments. Statistical analyses in (A) to (D) were performed by ANOVA followed by a Bonferroni post‐hoc test. Statistical analyses in (E) and (F) were analyzed using Student's *t* test.

CCK‐8 and clone formation assays were utilized to measure the proliferative capacity of cells. The chart form CCK‐8 assay showed that the absorbance values of MCF‐7 and MDA‐MB‐231 cells with CHPF depletion were significantly reduced compared to the si‐con or control (non‐transfected cells) group at 48 and 72 h (*P* < 0.01) (Fig. 3A,B). By contrast, MCF‐7 and MDA‐MB‐231 cells with CHPF overexpression revealed an enhanced cell viability compared to the vector group or control group (*P* < 0.01) (Fig. 3C,D). As for the clone formation assay, similar trends were observed. There were less visible colonies in si‐CHPF transfected MCF‐7 cells (150.00 ± 29.51) compared to in the si‐con group (358.33 ± 36.5; *P* < 0.05) (Fig. 3E), whereas the number of colonies of MDA‐MB‐231 cells transfected with pcDNA3.1‐CHPF (380.67 ± 47.17) was three‐fold greater compared to the vector group (123.33 ± 30.89; *P* < 0.01) (Fig. 3F). All of these results demonstrated that CHPF could promote BRCA cell viability.

**Fig. 4:**
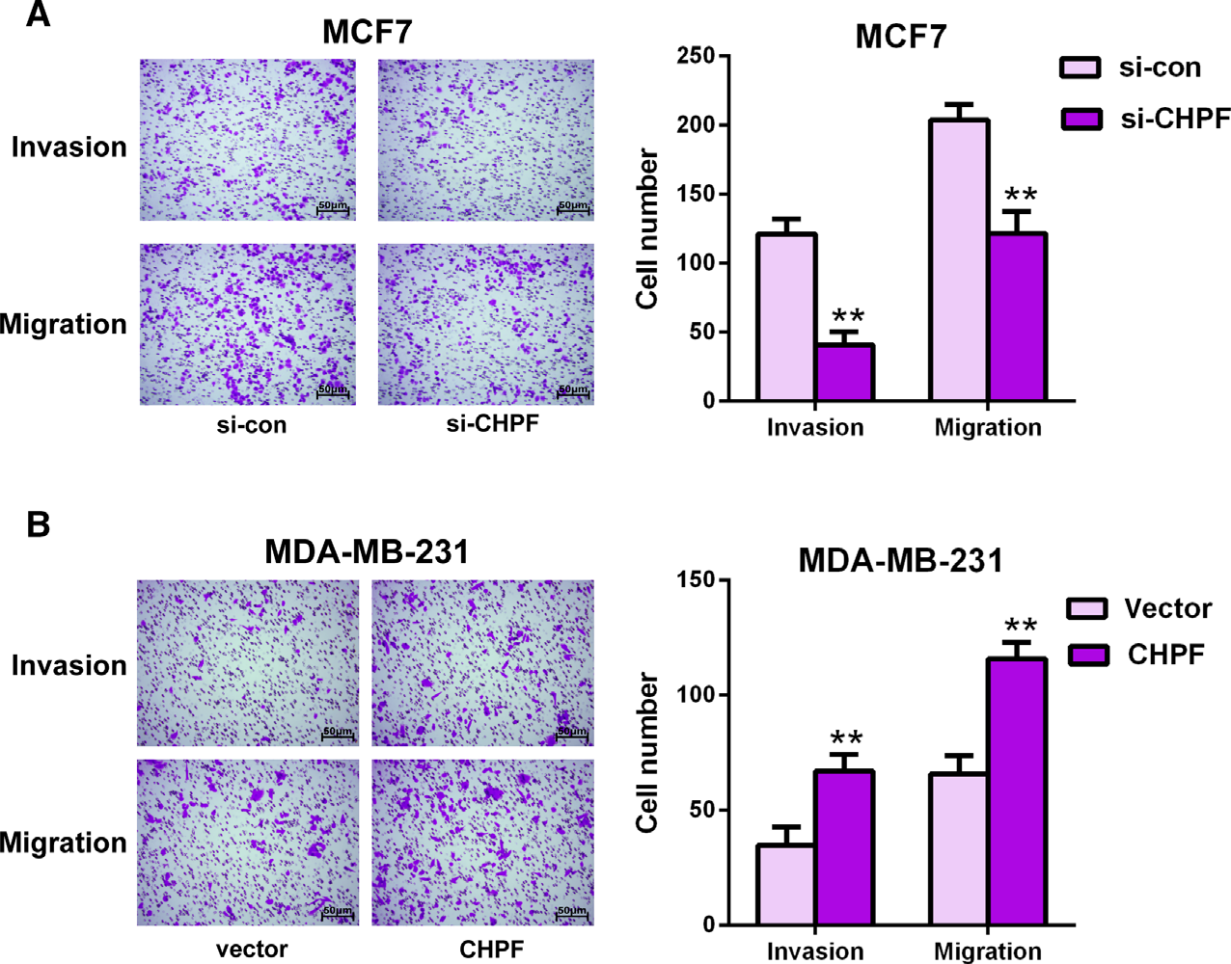
CHPF expression influenced BRCA cell invasion and migration. A transwell assay was used to evaluate the number of invaded and migrated of MCF7 (A) cells after transfection with si‐CHPF and si‐con. (B) MDA‐MB‐231 cells were transfected with pcDNA3.1‐CHPF and pcDNA3.1. **P < 0.01 vs. si‐con group or. vector group. Data are presented as the mean ± SD for three independent experiments. Statistical analyses were performed using Student's *t*‐test. Scale bar = 50 μm.

To determine whether CHPF contributes to BRCA cell invasiveness and motility, a transwell assay was conducted. The results shown in Fig. 4A indicated that the invasive and migratory capacities were both significantly decreased in CHPF‐depleted MCF7 cells compared to the si‐con group (*P* < 0.01). On the other hand, the roles of CHPF overexpression in MDA‐MB‐231 cells were also tested. As expected, overexpression of CHPF notably elevated the invasive and migratory abilities of BRCA cell compared to the vector group (*P* < 0.01) (Fig. 4B). Taken together, these data suggest that CHPF contributes to BRCA cell proliferation, invasion and migration and may act as a tumor promoter in BRCA.

### CHPF is associated with the levels of EMT‐related markers and MMP2

**Fig. 5:**
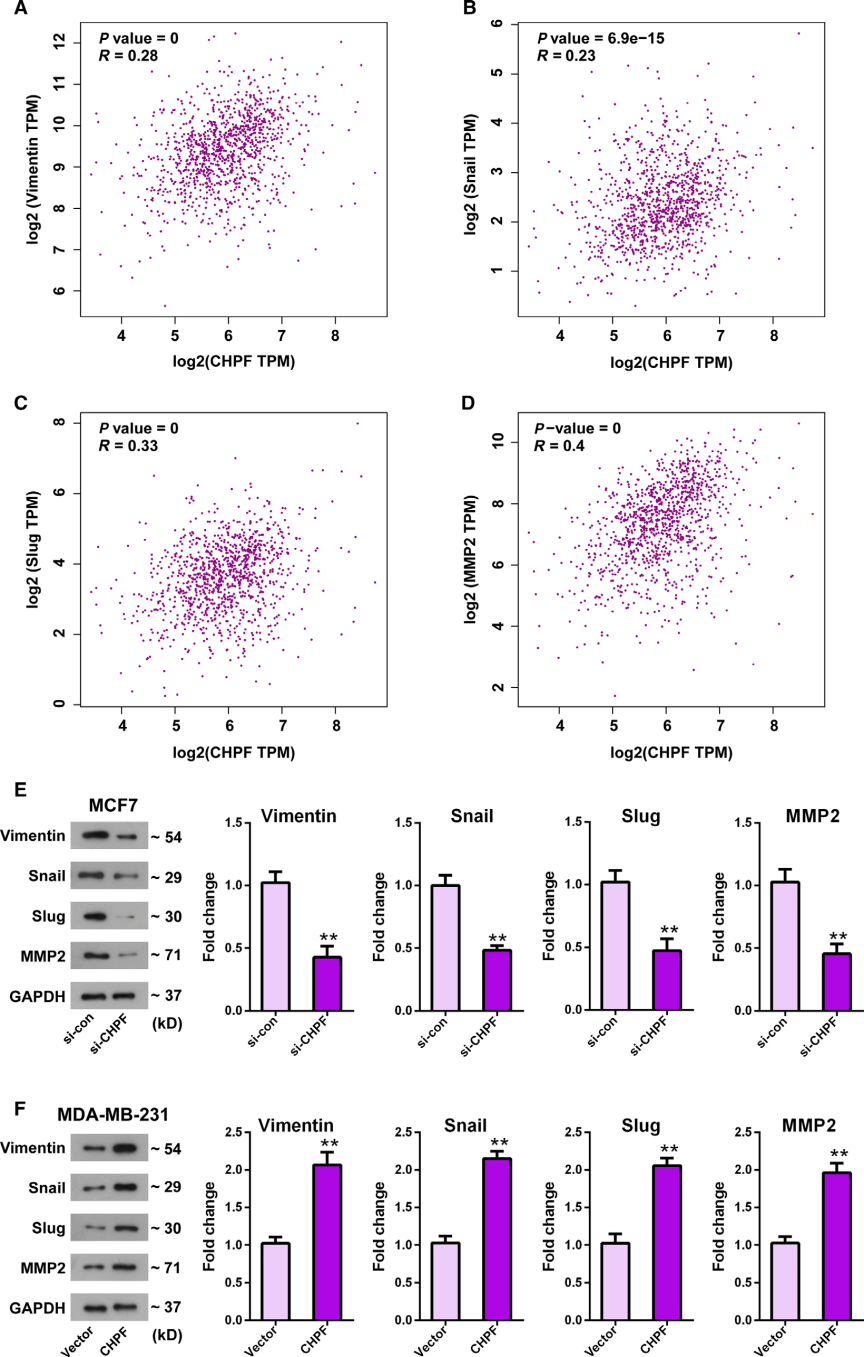
CHPF is associated with EMT relative markers and MMP2. (A–D) The GEPIA database was used to analyze the relationship between CHPF and metastatic markers, including vimentin, Snail, Slug and MMP2. (0 < *r* < 1, Pearson correlation coefficient). (E, F) Effects of CHPF depletion (E) or overexpression (F) on motion‐related markers in MCF7 (E) or MDA‐MB‐231 (F) cells were determined by western blotting. **P < 0.01 vs. si‐con group or vector group. Data are presented as the mean ± SD for three independent experiments. Statistical analyses were performed using Student's *t* test.

To determine the mechanism underlying the action of CHPF in BRCA, we used the GEPIA database to analyze the relevance between CHPF expression and transfer‐related molecules. As shown in Fig. 5A–C, CHPF expression was positively correlated with EMT relative marker, including vimentin, Snail1 and Slug (0 < *r* < 1, *P* < 0.01). Interestingly, a positive relationship between CHPF expression and MMP2 with a Pearson score of 0.4 was presented (*P* < 0.01) (Fig. 5D). Based on these findings, western blotting was performed to verify the protein levels of EMT‐related markers and MMP2 after altering the expression of CHPF. As shown in Fig. 5E, the results demonstrated that downregulation of CHPF significantly reduced the levels of vimentin, Snail1 and Slug, as well as MMP2 (*P* < 0.01) (Fig. 5E). However, upregulation of CHPF significantly increased the levels of EMT relative markers and MMP2 (*P* < 0.01) (Fig. 5F). In sum, these outcomes implied that CHPF shows a positive correlation with the EMT process and MMP2 expression.

### GSEA

To further explore the biological function of CHPF in BRCA, we used GSEA to predict the potential signaling pathways. The results shown in Table S1 suggested that increased CHPF expression was positively correlated with the tumor‐related signaling pathways, including ECM receptor interaction, transforming growth factor‐β signaling pathway and Wnt signaling pathway (*P* < 0.05).

## Discussion

Despite tremendous advances in cancer research, BRCA remains a major health burden and represents a current biomedical research priority. Worldwide, BRCA is the most common cancer in women, and its incidence and mortality are expected to rise significantly in the next few years [22]. Thus, it is very important to improve the diagnosis, treatment and prognosis of BRCA. In present study, we focused on investigating the biological role of CHPF in BRCA, as well as its effect on the motion‐related markers of BRCA cells.

A comprehensive analysis of the transcriptional expression of CHPF in BRCA was initially performed in the present study using TCGA and Oncomine databases. The data indicated that CHPF was upregulated in BRCA tissues compared to normal adjacent tissues, and its expression was significantly correlated with BRCA subtype. Our results are in accordance with the results of previous studies reporting that CHPF was upregulated in NSCLC, glioblastoma, colorectal cancer and laryngeal cancer [15, 16, 23, 24]. All of these findings provide evidence suggesting that CHPF may play a role in the progression of BRCA. In the next step, we found that the expression of CHPF was highly expressed in stage III + IV or metastasis tissues compared to stage I + II or non‐metastasis tissues, respectively. Moreover, we assessed the correlation between CHPF expression and the survival time in patients with BRCA, and found that patients with high expression of CHPF exhibited a poorer overall survival, DFS, DSS and PFS, which is in agreement with a previous study reporting that elevated CHPF expression resulted in a worse overall survival in lung cancer patients [25]. All of these data suggest that elevated CHPF expression may be related to poor overall survival in BRCA patients.

Furthermore, we investigated the role of CHPF knockdown or overexpression on BRCA cell malignant behaviors and found that MCF7 cells with depletion of CHPF demonstrated a powerful inhibitory effect with respect to proliferation, invasion and migration. Inversely, upregulation of CHPF in MDA‐MB‐231 cells showed the opposite results. Consistently, previous studies reported that downregulation of CHPF could significantly suppress lung adenocarcinoma cell proliferation, apoptosis and the cell cycle [15, 17]. Moreover, it has been reported that CHPF could promote glioma cell growth and restrain apoptosis [16]. In brief, these findings suggest that CHPF contributes to BRCA progression. It is noteworthy that, in our bioinformatics analysis, CHPF was not found to be significantly correlated with tumor stage using the data from TCGA database, which is somewhat in contrast to the effect of CHPF on BRCA cell proliferation that we detected in CCK‐8 and clone formation assays. We suspect that complex factors are involved such that establishing our own clinical dataset for further validation is necessary.

Metastasis remains the leading cause of death in BRCA patients [26]. Because CHPF is closely related to the invasiveness and motility of BRCA cells, the question arises of how it works. Based on online analysis, we found that CHPF was associated with metastasis relative markers vimentin, Snail1 Slug and MMP2. Vimentin is a representative mesenchymal marker of EMT with respect to maintaining cytoskeletal integrity [27]. Snail1 and Slug (also known as Snail2) are EMT transcription factors that could promote tumor cell progression by promoting invasion [28]. As reported previously, EMT comprises a major mechanism for explaining metastasis events in BRCA [29] and MMP2 was found to be a promoter and mediator with respect to participating in the pathogenic EMT process in the breast [30, 31]. Furthermore, multiple factors are reported to be related to BRCA progression via regulating EMT relative biomarkers [32, 33, 34]. Thus, we adopted western blotting to determine the effect of CHPF on EMT relative markers and MMP2, which have not been reported previously. Our data showed that knockdown of CHPF led to a significant reduction on the protein levels of vimentin, Snail1, Slug and MMP2, whereas overexpression of CHPF resulted in a notable elevation of these markers levels. Thus, we infer that CHPF might facilitate BRCA cell invasiveness and motility by regulating EMT and MMP2 expression.

In conclusion, the present study demonstrates for the first time that CHPF is aberrantly upregulated in BRCA and associated with a poor overall survival. In addition, we also initially observed that a loss of CHPF suppressed BRCA cell invasive and migratory abilities by regulating EMT relative biomarkers and MMP2 expression. The findings of the present study provide the basis for the targeted application of CHPF in BRCA.

## Conflict of interests

The authors declare that they have no conflicts of interest.

## Author contributions

YL, YQW and LZH conceived and designed the study. YL, HG, LF, DM and YJX acquired the data and also performed the experiments and statistical analysis. YL, HG and LF analyzed and interpreted the data. YL and YQW drafted and edited the manuscript. The authors declare that all data were generated in‐house and that no paper mill was used. All authors approved the final version of the manuscript submitted for publication.

## Supporting information


**Fig. S1.** CHPF expression in breast carcinoma from TCGA Breast Statistics in the Oncomine platform.
**Fig. S2.** High expression of CHPF was correlated with DFS, DSS and PFS. (a‐c) Survival curve was plotted by the Kaplan–Meier method based on TCGA database.
**Table S1.** The results of GSEA based on the differentially expressed genes with respect to tumors with high CHPF vs. low CHPF in TCGA cohort.

## Data Availability

The data and material used in the present study are available from the corresponding author on reasonable request.
