# Supplementary material for: Chondroitin polymerizing factor promotes breast carcinoma cell proliferation, invasion and migration and affects expression of epithelial‐mesenchymal transition‐related markers
Source: FEBS Open Bio. 2021 Jan 7;11(2):423–34. doi: 10.1002/2211-5463.13062 (PMC7876491; doi:10.1002/2211-5463.13062)
Supplement: Supplementary file 1 — Fig. S1. CHPF expression in breast carcinoma from TCGA Breast Statistics in the Oncomine platform. Fig. S2. High expression of CHPF was correlated with DFS, DSS and PFS. (a‐c) Survival curve was plotted by the Kaplan–Meier method based on TCGA database. Table S1. The results of GSEA based on the differentially expressed genes with respect to tumors with high CHPF vs. low CHPF in TCGA cohort. [file FEB4-11-423-s001.docx]

**Supplementary figure 1** **CHPF expression in breast carcinoma from TCGA Breast Statistics in the Oncomine Platform.**





**Supplementary figure 2 High expression of CHPF was correlated with DFS, DSS, and PFS.**

(a-c) Survival curve was plotted by Kaplan-Meier method based on the TCGA database.

**
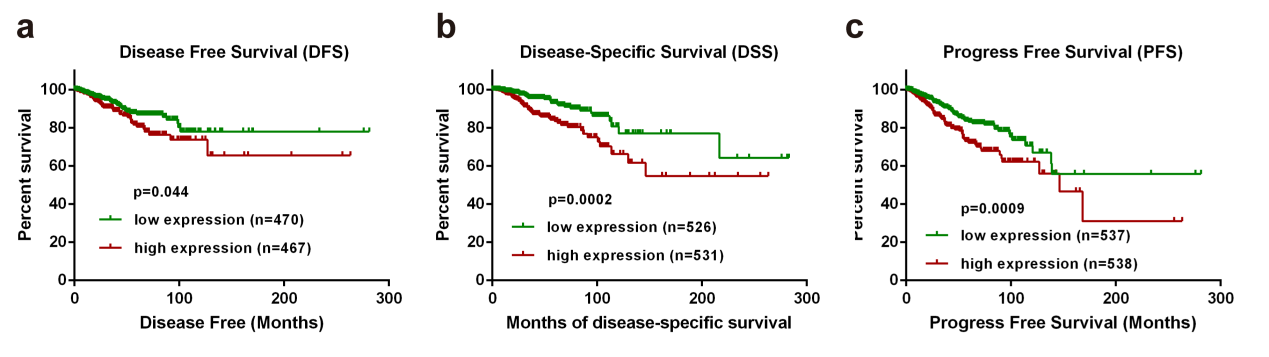
**

**Supplementary table 1 The results of GSEA based on the differentially expressed genes between tumors with high CHPF vs low CHPF in TCGA cohort.**

| **Number** | **NAME** | **SIZE** | **ES** | **NES** | **NOM p-val** | **FDR q-val** | **FWER p-val** |
| --- | --- | --- | --- | --- | --- | --- | --- |
| 1 | KEGG_EPITHELIAL_CELL_SIGNALING_IN_HELICOBACTER_PYLORI_INFECTION | 16 | 0.731329 | 2.066194 | 0 | 0.051695 | 0.033 |
| 2 | KEGG_VIBRIO_CHOLERAE_INFECTION | 12 | 0.827468 | 2.058118 | 0.001953 | 0.027655 | 0.036 |
| 3 | KEGG_AXON_GUIDANCE | 49 | 0.541565 | 2.042663 | 0.002198 | 0.022589 | 0.044 |
| 4 | KEGG_LYSOSOME | 21 | 0.695184 | 2.020984 | 0.00198 | 0.020544 | 0.054 |
| 5 | KEGG_HUNTINGTONS_DISEASE | 24 | 0.607286 | 2.019307 | 0 | 0.016776 | 0.055 |
| 6 | KEGG_OXIDATIVE_PHOSPHORYLATION | 12 | 0.782216 | 1.949454 | 0 | 0.02942 | 0.106 |
| 7 | KEGG_FOCAL_ADHESION | 74 | 0.550675 | 1.921707 | 0.004175 | 0.031019 | 0.127 |
| 8 | KEGG_ECM_RECEPTOR_INTERACTION | 38 | 0.643609 | 1.896138 | 0.00432 | 0.036756 | 0.162 |
| 9 | KEGG_FC_GAMMA_R_MEDIATED_PHAGOCYTOSIS | 22 | 0.565217 | 1.861336 | 0.001953 | 0.04527 | 0.218 |
| 10 | KEGG_PYRIMIDINE_METABOLISM | 23 | 0.606032 | 1.792662 | 0.011928 | 0.077293 | 0.349 |
| 11 | KEGG_REGULATION_OF_ACTIN_CYTOSKELETON | 62 | 0.449497 | 1.780913 | 0.00211 | 0.078295 | 0.381 |
| 12 | KEGG_NEUROTROPHIN_SIGNALING_PATHWAY | 19 | 0.549167 | 1.752569 | 0.018109 | 0.092453 | 0.446 |
| 13 | KEGG_N_GLYCAN_BIOSYNTHESIS | 6 | 0.788019 | 1.732004 | 0.008639 | 0.104112 | 0.501 |
| 14 | KEGG_AMINO_SUGAR_AND_NUCLEOTIDE_SUGAR_METABOLISM | 6 | 0.832656 | 1.725521 | 0.003883 | 0.101319 | 0.518 |
| 15 | KEGG_GLYCOSAMINOGLYCAN_BIOSYNTHESIS_CHONDROITIN_SULFATE | 6 | 0.81782 | 1.722474 | 0.010225 | 0.09744 | 0.527 |
| 16 | KEGG_TGF_BETA_SIGNALING_PATHWAY | 24 | 0.518278 | 1.718941 | 0.010526 | 0.094794 | 0.538 |
| 17 | KEGG_SNARE_INTERACTIONS_IN_VESICULAR_TRANSPORT | 5 | 0.803793 | 1.698841 | 0.005725 | 0.106401 | 0.585 |
| 18 | KEGG_RNA_DEGRADATION | 7 | 0.747693 | 1.654826 | 0.020833 | 0.141333 | 0.694 |
| 19 | KEGG_ALZHEIMERS_DISEASE | 26 | 0.506482 | 1.638345 | 0.017893 | 0.151792 | 0.74 |
| 20 | KEGG_PARKINSONS_DISEASE | 10 | 0.620101 | 1.625778 | 0.031373 | 0.157032 | 0.763 |
| 21 | KEGG_NICOTINATE_AND_NICOTINAMIDE_METABOLISM | 7 | 0.720111 | 1.625415 | 0.014257 | 0.149814 | 0.764 |
| 22 | KEGG_COLORECTAL_CANCER | 12 | 0.561941 | 1.574925 | 0.032454 | 0.201449 | 0.84 |
| 23 | KEGG_WNT_SIGNALING_PATHWAY | 31 | 0.429744 | 1.566495 | 0.02444 | 0.202907 | 0.854 |
| 24 | KEGG_ERBB_SIGNALING_PATHWAY | 23 | 0.466119 | 1.565758 | 0.025048 | 0.195324 | 0.855 |
